# Spinal epidural lipomatosis: An unusual cause of relapsing and remitting paraparesis

**DOI:** 10.4103/1817-1745.76117

**Published:** 2010

**Authors:** Dinesh Rajput, Arun K. Srivastava, Raj Kumar

**Affiliations:** Department of Neurosurgery, Sanjay Gandhi Postgraduate Institute of Medical Sciences, Lucknow, Uttar Pradesh, India

**Keywords:** Idiopathic cord compression, myelopathy, paraparesis, spinal epidural lipomatosis

## Abstract

Epidural lipomatosis is a rare entity to cause spinal cord compression and neurological deficits. This is usually associated with excess of steroids in the body either because of endogenous source as in Cushings disease or exogenous intake as in some diseases like systemic lupus erythematosus, in some endocrinopathies or in morbid obesity. But in some cases no cause has been found. Such idiopathic cases of spinal epidural lipomatosis have also been reported. Here, we report a case of idiopathic spinal epidural lipomatosis with relapsing and remitting paraparesis which is quite unusual. Treatment depends upon neurological status of the patient. We operated the patient as he had significant neurological compromise and he improved significantly.

## Introduction

Spinal epidural lipomatosis is defined as pathological overgrowth of the normally presented extradural fat and often causes dural impingement. Symptomatic epidural lipomatosis was first described by Lee *et al*. in 1975 in a patient after renal transplantation. Subsequent reports on this rare clinical entity also implicated the administration of steroid as the major cause for spinal epidural lipomatosis.

## Case Report

**Figure 1:**
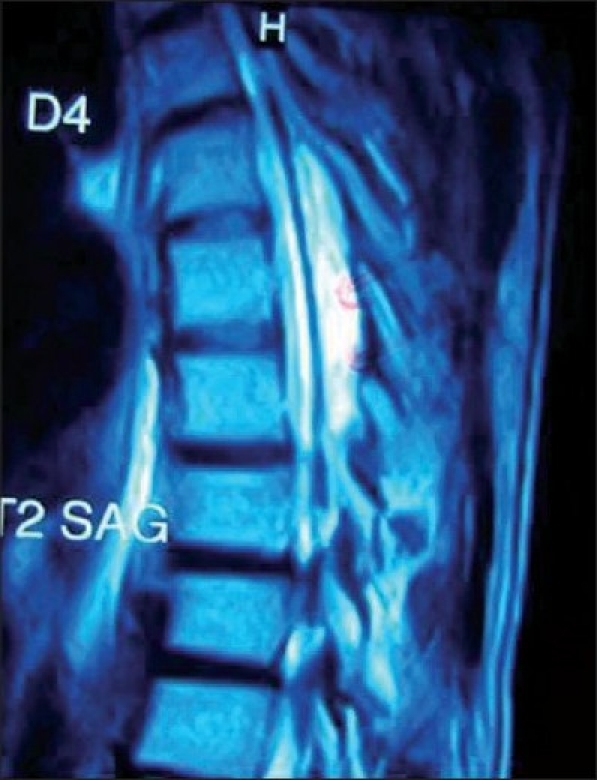
Midsagittal T2W1 image showing hyperintense lesion on posterior aspect of spinal canal of thickness 10 mm extending from D5 to D7 level

**Figure 2:**
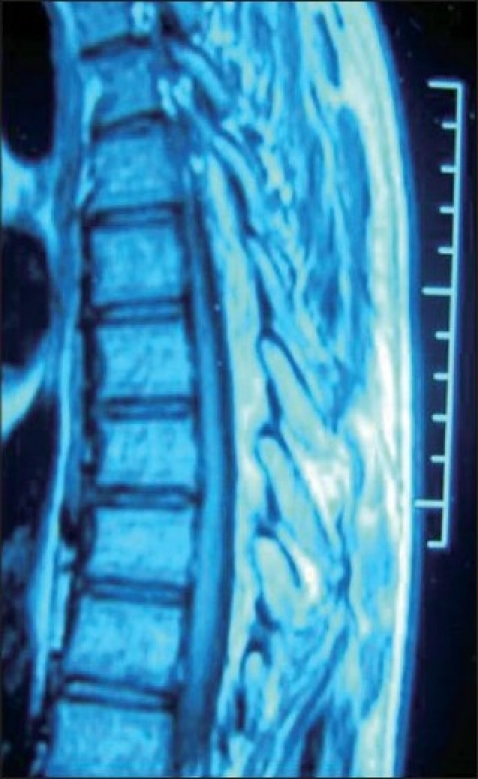
Midsagittal T1W1 image showing hyperintense lesion on posterior aspect of spinal canal of thickness 10 mm extending from D5 to D7 level

**Figure 3:**
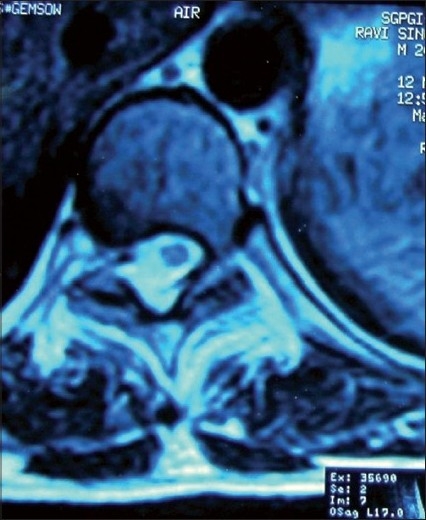
Axial T2W1 image showing hyperintense lesion on posterior and right lateral aspects of spinal canal

We report an 18-year-old male who presented to neurosurgery outpatient department with complaints of fluctuating paraparesis for 6 months. He noticed weakness of lower limbs when he woke up one morning; he failed to move his lower limbs but after half an hour, he started moving with persistent unsteadiness and difficulty in walking. This deficit also recovered the same evening. He remained asymptomatic for the next 4 months when he again developed ascending weakness of lower limbs along with spasticity, and in the next 3 months, he was not able to stand even with support. He developed retention of urine for which a Foley’s catheter was installed. He had constipation for 1 month. This patient denied history of trauma, fever, drug intake like steroids, antithyroid drugs, and infections. Physical examination revealed a non-obese patient [weight 65 kg, height 167 cm, body mass index (BMI) 23.30 kg/m^2^] without any evidence of Cushings syndrome or endocrinopathies. Systemic examination was normal. Neurological examination revealed increased tone in lower limbs with preserved muscle bulk. Power at hip and knee was 3/5 while at ankle and toes it was 0/5 (Medical Research Council grading) with brisk knee and ankle jerks. Bilateral planters were extensor. There was a graded sensory loss below D10 level. Local examination did not reveal any spinal deformity or tenderness. He was diagnosed to have compressive myelopathy in thoracic region. His hematological and biochemical investigations were normal. Cerebrospinal fluid (CSF) examination was also normal. X-ray spine was unremarkable, but magnetic resonance imaging (MRI) thoracic spine showed posterior epidural mass in dorsal spine extending from D5 to D7 level with maximum thickness of 10 mm and causing cord compression. This mass was homogeneously hyperintense in both T1W1 and T2W1 images suggestive of fat [Figures 1–3]. He was operated and lipoma with poor plane of cleavage was present. Histopathology was suggestive of lipoma. This patient was discharged and at follow-up of 6 months, he was able to walk with support.

## Discussion

Spinal epidural lipomatosis is characterized by abnormal deposition of unencapsulated fat in epidural space.[1] This condition is usually associated with excess of steroid levels either because of exogenous steroid administration, as in some diseases like systemic lupus erythematosis, or endogenous excess steroid secretion like in Cushings disease or in some other endocrinopathies like hypothyroidism or in morbid obesity.[1–3] In some cases where no such cause can be established, these are treated as idiopathic types.[4] Although spinal epidural lipomatosis is seen mostly in patients taking high doses of glucocorticoids (30 mg /d taken over several months), it also has been reported with steroid doses as low as 15 mg prednisone per day even for a short period of 4 months.[5,6] In Cushings syndrome, there occurs centripetal fat deposition, i.e., in regions of trunk, neck, faces and less commonly in mediastinum and popliteal fossa.[7] Fat deposition in epidural space, causing spinal cord compression is rare, which produces local pain and neurological deficits.[7] Neurologic deficits are in the form of compressive myelopathy, radiculopathy, cauda equina[4] and may present with complaints of muscle weakness, sensory loss, or abnormal reflexes.[8] Low backache may be present. Onset is usually gradual with slow progression of neurological features over several months, although rapid onset[9] and relapsing and remitting courses with sensory symptoms have also been reported.[10] Our case is unique in the manner in which the patient developed weakness of lower limbs which recovered over several hours, followed by progressive weakness of lower limbs after 4 months. The pathogenesis is probably multifactorial; venous stasis with thrombosis has been suggested as precipitin syndrome.[11] Such a course of disease mimics demyelinating disorders like multiple sclerosis.[10] Myelopathy is because of mechanical compression of the spinal cord and the vascular dysfunction caused by compression of the epidural blood vessels with venous engorgement.[10]

Males are affected more than females, with 75% of reported cases being males.[10–12] Thoracic spine is affected more followed by lumbar region, while cervical epidural lipomatosis has not been reported till now.[13,14]

MRI spine is diagnostic choice of imaging. It shows uniformly hyperintense fat collection in epidural space on both T1W1 and T2W1 sequences.[2] Circumferential compression of the thecal sac is referred to as “Y-sign”, and is pathognomic in lumbar axial imaging.[15] Spinal angiolipoma, primary and secondary spinal tumors and abscesses are important differential diagnoses which can be ruled out with contrast imaging.[2,11] Epidural fat thickness greater than 7 mm is diagnostic of spinal epidural lipomatosis.[10,11] In our patient, maximum thickness of fat was 10 mm but “Y-sign” was not evident because of asymmetrical deposition of fat [Figure 3].

Treatment depends upon severity of neurological deficits. Conservative treatment has been reported to be successful in many patients, i.e., weaning of exogenous steroids or treatment of Cushing disease and weight loss.[2,15] But our patient neither had obesity (BMI = 23.30 kg/m^2^) nor had excess steroids. Surgical intervention is indicated in severe progressive neurological deficits, which include decompressive laminectomy and resection of epidural adipose tissue.[1,2,4,10,11] Decompressive treatment is successful in majority of patients and they may have neurological recovery after surgical intervention. The prognosis of idiopathic spinal epidural lipomatosis is favorable after surgical intervention and no cases of recurrence have been reported following surgery.[1] Our patient was non-obese with no evidence of endocrinopathy and presented with relapsing and remitting course of paraparesis over 6 months, which is quite unusual.

To conclude, spinal epidural lipomatosis should be considered as differential diagnosis of epidural mass causing compressive myelopathy with relapsing and remitting course of paraparesis. It may be present even in the absence of endocrinopathies or in non-obese patients. MRI spine with contrast is diagnostic and early diagnosis is important as minor symptoms may be relieved on conservative treatment while advanced and progressive disease requires surgical intervention.
